# Novel Inhibitors of Carbonic Anhydrase I and II Identified by High Throughput Virtual Screening and Validated by X‐Ray Crystallography

**DOI:** 10.1002/cmdc.70391

**Published:** 2026-07-16

**Authors:** Asher L. Brandt, Joseph Venditto, Marta Ferraroni, Claudiu T. Supuran, Andrea Angeli

**Affiliations:** ^1^ Department of Chemistry University of Saint Joseph West Hartford Connecticut USA; ^2^ Department of Chemistry “Ugo Schiff” University of Florence Sesto Fiorentino Italy; ^3^ NEUROFARBA Department Sezione di Scienze Farmaceutiche University of Florence Florence Italy

**Keywords:** carbonic anhydrase, carboxylic acids, high throughput virtual screening, metalloenzyme, X‐ray crystallography

## Abstract

High‐throughput virtual screening (HTVS) enables rapid exploration of large chemical spaces to identify novel modulators of biologically relevant targets. In this study, we applied a large‐scale docking campaign to discover new inhibitors of the human Carbonic Anhydrase isoforms I and II. Starting from library of 28,000 compounds, iterative Glide HTVS, SP, and XP docking against hCA I and hCA II yielded 81 top‐ranked compounds for experimental evaluation. Among these, five compounds showed micromolar inhibition of hCA II, and one also inhibited hCA I. X‐ray crystallographic studies of two carboxylate‐based inhibitors (**1** and **2**) revealed distinct binding modes. Compound **1** directly coordinates the catalytic zinc ion, whereas compound **2** adopts two different binding poses: one anchored to the zinc‐bound water molecule and another within a surface‐accessible pocket near His64. The latter region, which modulates the proton‐shuttling mechanism essential for catalysis, represents a less‐explored site for inhibitor development. Overall, these findings validate our HTVS workflow and provide a foundation for the structure‐guided optimization of carboxylate scaffolds as carbonic anhydrase inhibitors.

## Graphical abstract

High‐throughput virtual screening identifies novel carbonic anhydrase II ligands from large chemical libraries, enabling rapid hit discovery. Structural validation by X‐ray crystallography confirms the predicted binding modes and reveals key protein–ligand interactions. The combined computational and experimental workflow demonstrates an efficient strategy for accelerating the discovery of carbonic anhydrase inhibitors.

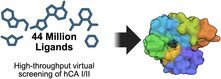

## 1 Introduction

The use of computational tools in drug discovery has been rapidly expanding in both industry and academia [1, 2, 3]. This growth is driven by several factors: (1) the increasing number of solved crystal structures in the Protein Data Bank [4] (PDB), (2) the accessibility of powerful CPUs and GPUs, (3) the availability of user‐friendly molecular modeling programs such as Schrödinger Maestro [5], OpenEye [6], AutoDock Vina [7], and GROMACS [8], and (4) the development of massive chemical screening libraries like ZINC22 [9]. Together, these advances make it possible to perform high‐throughput virtual screening (HTVS) on the binding pocket of nearly any protein with a solved structure. Remarkably, with readily available CPUs such as the Ryzen 9 5950X and GPUs like the RTX 3090, such large‐scale computational tasks can now be carried out efficiently on standard personal workstations [1]. Using this setup, on the order of one million ligands can be screened within several days, greatly increasing the diversity of chemical scaffolds that can be evaluated as potential binders. The present study focuses on carbonic anhydrases (CAs, E.C. 4.2.1.1), a family of metalloenzymes usually containing a catalytic zinc ion [10, 11, 12]. The human genome encodes 15 isoforms, 12 of which are catalytically active and 3 of which are CA‐related proteins (CARPs) lacking enzymatic activity [13, 14]. These enzymes catalyze the reversible hydration of carbon dioxide to bicarbonate and hydrogen ions, thereby regulating pH and ion transport. CA I, primarily expressed in erythrocytes and gastrointestinal tissue, has relatively low catalytic efficiency (kcat ≈ 2 × 10^5^ s^−1^), and its isolated inhibition has no established therapeutic role; however, inhibition of erythrocyte isoforms (CA I and CA II) can have important physiological consequences [15, 16]. Indeed, these enzymes are essential for maintaining systemic acid–base balance by catalyzing the rapid hydration of CO_2_ in red blood cells, thereby ensuring efficient CO_2_ transport from peripheral tissues to the lungs. When their activity is reduced, CO_2_ handling becomes less efficient and bicarbonate buffering capacity declines, ultimately promoting systemic metabolic acidosis. Such disorders in acid–base homeostasis are frequently associated with fatigue, reduced exercise tolerance, headache, and malaise, side effects that are widely documented for nonselective CA inhibitors. These symptoms arise partly because acidosis increases respiratory drive and energy demand, further contributing to the sensation of tiredness. Therefore, avoiding strong inhibition of erythrocyte CAs is a key consideration in the development of isoform‐selective CA inhibitors [17, 18]. CA II, in contrast, is widely expressed (erythrocytes, kidneys, eyes, gastrointestinal tract, and central nervous system) and is among the fastest known enzymes (kcat ≈ 1 × 10^6^ s^−1^). Its inhibition has well‐established therapeutic applications, including the treatment of glaucoma, epilepsy, altitude sickness, and edema through its diuretic effects [19, 20]. Despite extensive study, the design of selective CA inhibitors remains challenging due to the high structural similarity among isoforms. By leveraging modern HTVS approaches with large chemical libraries, this study aims to explore novel scaffolds capable of selectively modulating CA I and CA II activity.

## 2 Results and Discussion

**FIGURE 1:**
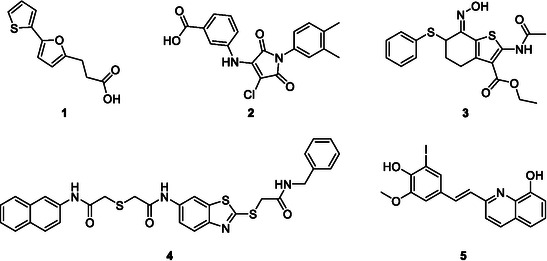
Chemical structures of active CAIs **1–5** from the 81 candidate compounds.

Initial virtual screening using Glide HTVS and Glide SP, followed by Glide XP docking with expanded sampling against hCA I (8RLO) and hCA II (4QY3), reduced the virtual libraries to 15,000 top‐ranked ligands for 8RLO and 13,000 for 4QY3. These 28,000 ligands were then grouped by scaffold, and for each chemotype we selected the highest‐scoring, commercially available representative. This procedure yielded a final set of 81 candidate compounds (52 from the 8RLO screen and 29 from the 4QY3 screen), all of which were purchased and evaluated experimentally (Table S1 and Figure 1). Among these, five compounds (**1–5**, Figure 1) exhibited inhibition against the cytosolic hCA I and hCA II.

These isoforms have been used in the assay due to the fact that the cytosolic hCAs I and II are widespread house‐keeping enzymes, involved in a host of physiologic processes. The obtained results are summarized in Table 1, alongside the standard inhibitor acetazolamide (**AAZ**), for comparison.

**TABLE 1:** Inhibition data of human CA isoforms I and II with compounds **1–5** and **AAZ** by a stopped‐flow CO_2_ hydrase assay [21].

*K*_I_, μM^a^			
Cmp	hCA I	hCA II	Selectivity index (SI) hCA II/hCA I
**1**	55.2	97.2	1.76
**2**	>100	75.3	>0.75
**3**	>100	92.1	>0.91
**4**	>100	74.7	>0.75
**5**	>100	75.0	>0.75
**AAZ**	0.250	0.012	0.048

a Mean from three different assays, by a stopped flow technique (errors were in the range of ±5%–10% of the reported values).

**FIGURE 2:**
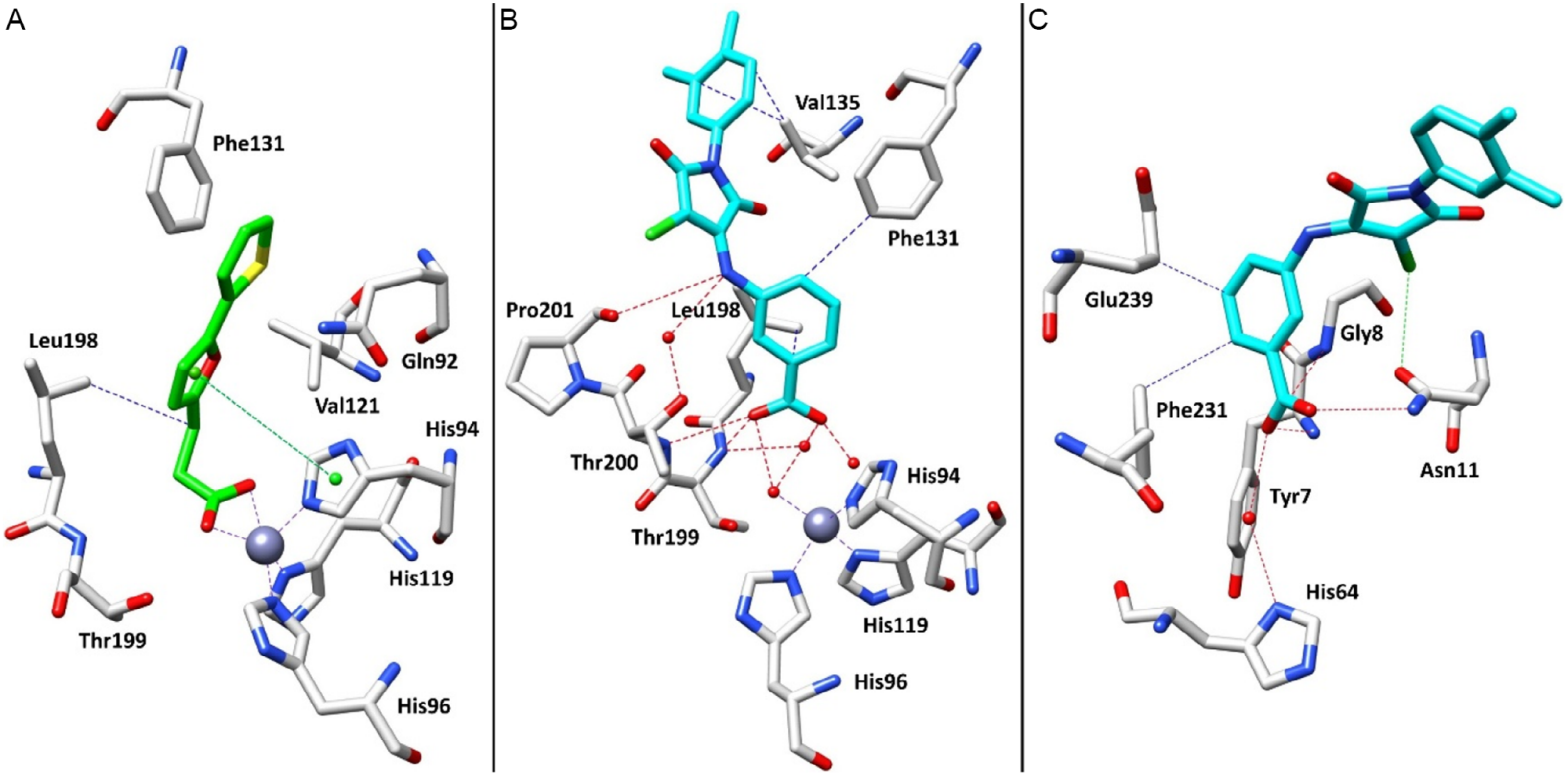
(A) X‐ray crystal structure of hCA II in complex with compound **1** (shown in green; PDB ID: 9TFR). (B) Crystal structure of hCA II with compound **2** bound within the active site and (C) compound **2** bound outside the active site (cyan; PDB ID: 9TF8). Key amino acid residues involved in ligand binding are highlighted. The catalytic Zn^2+^ ion is depicted as a gray sphere. Noncovalent interactions are indicated as follows: van der Waals contacts (blue), hydrogen bonds, and water bridges interactions (red), and *π*–*π* stacking interactions (green).

Among the five tested compounds, only inhibitor **1** displayed inhibitory activity against hCA I, with an inhibition constant of 55.2 μM. In contrast, all five compounds inhibited hCA II, with *K*_I_ values in the high‐micromolar range (74.73–97.22 μM). Notably, compounds **2–4** showed selectivity toward hCA II, as they did not inhibit hCA I under the same conditions. In order to better understand how carboxylate inhibitors interact with hCA II, we examined two different carboxylic acids by X‐ray crystallography. Carboxylates are known to adopt multiple binding modes within the hCA II active site [22, 23, 24], and our structures confirm this variability. For compound **1**, the inhibitor binds directly to the catalytic Zn(II) ion through its carboxylate group, with Zn–O distances of 1.9 and 2.4 Å. In addition, the inhibitor aliphatic chain engages in a hydrophobic contact with Leu198, and its furan ring forms a *π*‐stacking interaction with His94 (Figure 2A). Apart from these interactions, the molecule does not form additional interactions with the enzyme, which likely explains its relatively low inhibitory activity. Moving on, the hCA II‐**2** complex reveals one well‐defined inhibitor molecule occupying the active site (Figure 2B).

Its carboxylate establishes a hydrogen bond with the Zn(II)‐bound water molecule (2.6 Å) and the same oxygen atom participates in two additional hydrogen bonds with the backbone amide groups of Thr200 and Thr199. The second carboxylate oxygen interacts more weakly with the catalytic water (2.9 Å) and forms an additional water‐mediated contact with Thr199. The aromatic benzoate portion of the inhibitor participates in hydrophobic interactions with Leu198 and Phe131, and the secondary amine contributes a direct hydrogen bond to Pro201 as well as a water–bridged interaction with Thr200. This binding mode differs from that of compound **1**, likely due to steric hindrance imposed by the *meta*‐substituted benzoate scaffold when positioned within the active‐site cavity [25]. A noteworthy feature of the hCA II‐**2** structure is the presence of a second inhibitor molecule bound within a surface‐accessible cavity bordered by Gly6, Tyr7, Gly8, Asn11, His64, Phe231, Asn232, and Glu239 (Figure 2C). Multiple polar contacts stabilize the molecule in this region: one oxygen atom of the carboxylate accepts hydrogen bonds from the backbone nitrogen atoms of Tyr7 and Gly8, and a water‐mediated interaction holds His64 in its “outward” conformation. This configuration is consistent with previously described surface‐binding events involving carboxylic acids, where a restrained His64 orientation interferes with the proton‐shuttling mechanism required for catalytic turnover [26]. The other carboxylate oxygen forms a hydrogen bond with Asn11, and several additional contacts stabilize the aromatic scaffold. In particular, the phenyl ring establishes van der Waals contacts with Phe231 and Glu239, while the chlorine atom on the pyrroledione ring participates in a halogen bond interaction with Asn11. For both inhibitors, the terminal phenyl ring shows weak electron density (Figure S1), consistent with limited interactions with nearby residues, allowing it to remain flexible.

## 3 Conclusion

HTVS followed by experimental evaluation identified five micromolar inhibitors of the cytosolic isoform hCA II. One of them, compound 1 showed also micromolar inhibition against hCA I. X‐ray crystallographic analysis of two carboxylate compounds revealed distinct binding modes, including a secondary pocket near His64 not widely exploited in previous CA inhibitor design. These findings suggest new opportunities for structure‐based optimization of carboxylate scaffolds. Future work will focus on improving potency and selectivity through targeted modification of substituents occupying the hydrophobic pocket and surface‐accessible cavity.

## 4 Experimental Section

### 4.1 Chemicals Compounds

All 81 selected compounds were ordered from Specs (Zoetermeer, The Netherlands) via the MolPort online catalog and used without further purification.

### 4.2 Computational Resources

All calculations were performed on a custom‐built workstation equipped with an ASUS Strix B550‐F Gaming AM4 ATX motherboard, 128 GB Corsair Vengeance RGB Pro DDR4 memory (4 × 32 GB modules), a Samsung 990 Pro 4 TB NVMe SSD, an AMD Ryzen 9 5950X 16‐core/32‐thread processor, and an NVIDIA RTX 3090 Founders Edition GPU, powered by a Corsair RM850x fully modular power supply. The system ran Linux Mint 22.1, and all simulations were conducted using Schrödinger Maestro 2024‐2.

### 4.3 High Throughput Virtual Screen

Protein structures from the PDB (8RLO, human CA I in complex with veralipride, and 4QY3, human CA II bound to an ortho‐substituted benzoic acid) were prepared in Schrödinger Maestro 2024‐2 using the Protein Preparation Wizard to correct protonation states, add missing hydrogens, and perform local minimization. A library of 44 million ligands was downloaded from the ZINC22 database, imported into Maestro, and processed with LigPrep to assign the correct protonation states at physiological pH (7.4).

All 44 million ligands were docked into the binding sites of 8RLO and 4QY3, using the respective cocrystallized ligands as benchmarks. In this initial screening, approximately 5 million ligands scored more favorably than the reference ligand in 8RLO and 3 million ligands in 4QY3. These subsets were refined using Glide SP, yielding 135,000 ligands for 8RLO and 85,000 ligands for 4QY3 with more favorable docking scores than their cocrystallized ligands. The remaining ligands were then subjected to Glide XP docking with expanded sampling, reducing the pools to 15,000 ligands for 8RLO and 13,000 ligands for 4QY3.

The final ligand sets were clustered by chemical scaffold, producing 550 unique scaffolds for 8RLO and 335 scaffolds for 4QY3. From each scaffold, the compound with the most favorable docking score was selected and searched in the MolPort database, resulting in the purchase of 52 compounds for 8RLO and 29 compounds for 4QY3 for subsequent experimental evaluation.

### 4.4 CA Inhibition

CA‐mediated CO_2_ hydration was measured using a stopped‐flow spectrophotometer (Applied Photophysics) [21]. Phenol red (0.2 mM) served as the pH indicator, monitored at 557 nm. The assay was conducted in 20 mM HEPES buffer (pH 7.4) supplemented with 20 mM Na_2_SO_4_ to maintain constant ionic strength. Initial reaction rates were recorded over 10–100 s following CO_2_ addition. Substrate concentrations ranged from 1.7 to 17 mM to determine kinetic and inhibition parameters [27] and enzyme concentrations were maintained between 5 and 12 nM. For each inhibitor, at least six independent traces corresponding to the initial 5%–10% of the reaction progress were analyzed to calculate initial velocities. Background uncatalyzed reaction rates were measured under identical conditions and subtracted from the observed enzymatic rates. Inhibitor stock solutions (0.1 mM) were prepared in distilled deionized water with 10% DMSO and serially diluted with assay buffer to final concentrations as low as 0.01 nM. Prior to measurement, enzyme and inhibitor solutions were incubated together for 15 min at room temperature to allow equilibration of the enzyme–inhibitor complex. Inhibition constants (*K*_I_) were determined by nonlinear regression analysis using GraphPad Prism and calculated according to the Cheng–Prusoff equation. Reported values represent the mean of at least three independent experiments. Recombinant CA isoforms were produced in‐house as previously described [28, 29, 30].

### 4.5 Crystallization and X‐Ray Data Collection

Crystals of human CA II were grown by the hanging‐drop vapor diffusion technique in 24‐well Linbro plates. Drops were prepared by mixing 2 μL of a 10 mg/mL hCA II solution in 20 mM Tris–HCl buffer (pH 8.0) with 2 μL of reservoir solution containing 1.5 M sodium citrate and 0.1 M Tris buffer (pH 8.0). The drops were equilibrated against the same reservoir solution at 296 K. Protein–ligand complexes were generated by soaking native hCA II crystals in mother liquor supplemented with the inhibitors at a final concentration of 10 mM for 48 h. Prior to data collection, crystals were cryoprotected by adding 15% (v/v) glycerol to the mother liquor and flash‐cooled in liquid nitrogen at 100 K. Diffraction data for the CA II–**1** complex were collected at the ID30A‐1/MASSIF‐1 beamline (ESRF, Grenoble, France) using synchrotron radiation at a wavelength of 0.965459 Å and a DECTRIS PILATUS4 X 4 M detector. Data for the CA II–**2** complex were acquired at the XRD2 beamline of Elettra Synchrotron (Trieste, Italy) with a wavelength of 1.000 Å and a DECTRIS Pilatus 6 M detector. In both cases, diffraction images were processed and scaled using the XDS software package [31]. Detailed data collection and processing statistics are provided in the Supporting Information.

### 4.6 Structure Determination

Initial phases were obtained by molecular replacement using the hCA II structure (PDB ID: 4FIK) using Refmac5 [32] with solvent molecules and heteroatoms removed. A randomly selected 5% subset of reflections was omitted from refinement to calculate the Rfree value. Difference electron density maps (|Fo – Fc|) clearly revealed the presence of the inhibitor molecules, which were modeled with full occupancy. Refinement was carried out using standard positional and isotropic B‐factor refinement protocols, interspersed with manual model building in COOT [33]. Model validation and quality assessment were performed using COOT and RAMPAGE [34]. Crystallographic parameters and refinement statistics are reported in the Electronic Supplementary Information. Final atomic coordinates have been deposited in the PDB under accession codes 9TFR and 9TF8. Structural figures were prepared using UCSF Chimera [35].

## Conflicts of Interest

The authors declare no conflicts of interest.

## Supporting information

Supplementary Material

## Data Availability

The data that support the findings of this study are available from the corresponding author upon reasonable request.
