# Supplementary material for: Novel Inhibitors of Carbonic Anhydrase I and II Identified by High Throughput Virtual Screening and Validated by X‐Ray Crystallography
Source: ChemMedChem. 2026 Jul 16;21(14):e70391. doi: 10.1002/cmdc.70391 (PMC13375402; doi:10.1002/cmdc.70391)
Supplement: Supplementary file 1 — Supplementary Material [file CMDC-21-e70391-s001.pdf]

Supplementary Material for

Novel Inhibitors of Carbonic Anhydrase I and II Identified by High Throughput Virtual Screening and Validated by X-ray Crystallography.

Asher L. Brandt[a], Joseph Venditto[a], Marta Ferraroni[b], Claudiu T Supuran[c], Andrea Angeli[c*]

[a] Department of Chemistry, University of Saint Joseph, West Hartford CT, 06117 USA

[b] Department of Chemistry "Ugo Schiff", University of Florence, Via della Lastruccia 3-13, I-50019, Sesto Fiorentino, Italy

[c] NEUROFARBA Department, Sezione di Scienze Farmaceutiche, University of Florence, Via Ugo Schiff 6, 50019, Sesto Fiorentino, Florence, Italy.

*Corresponding author: andrea.angeli@unifi.it

Index

Inhibition data of human CA isoforms I and II with screening compounds *S2*

Summary of Data Collection and Atomic Model Refinement Statistics for hCAII *S3*

Figure S1 *S4*

Table S1. Inhibition data of human CA isoforms I and II with screening compounds by a stopped-flow CO_2 hydrase assay

K_I (µM)* (% inhibition at 100 µM)					
**Cmp**	**hCA I**	**hCAII**	**Cmp**	**hCA I**	**hCAII**
**Molport-001-791-076**	11%	26%	**Molport-001-821-451**	22%	38%
**Molport-000-014-344**	17%	20%	**Molport-001-672-458**	19%	30%
**Molport-001-944-304**	25%	26%	**Molport-002-180-726**	22%	44%
**Molport-000-723-605**	24%	35%	**Molport-001-966-687**	26%	39%
**Molport-001-676-062**	24%	7%	**Molport-000-211-228**	9%	21%
**Molport-002-192-741**	19%	10%	**Molport-000-500-790**	16%	43%
**Molport-000-661-504**	4%	22%	**Molport-002-695-951**	12%	25%
**Molport-002-794-819**	14%	33%	**Molport-019-769-567**	13%	28%
**Molport-000-995-891**	27%	33%	**Molport-002-793-095**	10%	13%
**Molport-002-678-798**	39%	49%	**Molport-009-758-115**	11%	23%
**Molport-002-213-847**	10%	2%	**Molport-019-771-184**	11%	39%
**Molport-002-794-946**	34%	27%	**Molport-019-787-167**	5%	38%
**Molport-000-650-875**	28%	21%	**Molport-001-623-837**	6%	46%
**Molport-001-955-639**	37%	23%	**Molport-002-795-615**	30%	21%
**Molport-001-958-886**	11%	21%	**Molport-001-567-869**	35%	10%
**Molport-002-674-719**	20%	17%	**Molport-002-833-826**	29%	22%
**Molport-000-650-880**	17%	14%	**Molport-002-644-724**	15%	11%
**Molport-002-177-665**	21%	15%	**Molport-002-008-490**	21%	14%
**Molport-001-487-334**	24%	18%	**Molport-006-838-227**	32%	39%
**Molport-002-550-578**	14%	24%	**Molport-000-946-550**	29%	9%
**Molport-002-794-378**	14%	21%	**Molport-002-826-580**	30%	26%
**Molport-001-991-122**	14%	10%	**Molport-001-573-189**	40%	34%
**Molport-001-925-400**	25%	11%	**Molport-002-221-291**	38%	43%
**Molport-000-417-160**	22%	17%	**Molport-002-354-605**	26%	18%
**Molport-000-152-673**	1%	17%	**Molport-000-854-370**	10%	23%
**Molport-000-385-132**	11%	39%	**Molport-002-004-874**	13%	29%
**Molport-000-629-475**	15%	8%	**Molport-001-532-224**	23%	46%
**Molport-000-889-221**	2%	27%	**Molport-000-682-537**	15%	40%
**Molport-001-931-558**	8%	1%	**Molport-002-238-168**	41%	46%
**Molport-002-510-785**	26%	32%	**Molport-003-059-323**	26%	19%
**Molport-000-654-190**	25%	36%	**Molport-002-835-845**	17%	29%
**Molport-000-653-683**	22%	41%	**Molport-001-940-806**	14%	30%
**Molport-000-742-906**	22%	7%	**Molport-000-690-803**	9%	26%
**Molport-001-847-891**	11%	17%	**Molport-002-216-390**	23%	38%
**Molport-001-894-025**	27%	31%	**Molport-002-793-026**	19%	34%
**Molport-002-164-691**	25%	16%	**Molport-000-683-322**	6%	15%
**Molport-000-482-003**	3%	29%	**Molport-000-630-468**	24%	30%
**Molport-006-779-995**	28%	19%	**Molport-001-023-108**	31%	32%

* Mean from 3 different assays, by a stopped flow technique (errors were in the range of ± 5-10 % of the reported values).

Table S2. Summary of Data Collection and Atomic Model Refinement Statistics for hCAII

	**hCAII + 1**	**hCAII + 2**
PDB ID	9TFR	9TF8
Wavelength (Å)	0.965459	1.00
Space Group	P21	P21
Unit cell (a, b, c, α, β, γ)(Å,°)	42.42, 41.43, 72.23<br>90.00, 104.38, 90.00	42.29, 41.38, 72.15<br>90.00, 104.39, 90.00
Limiting resolution (Å)	41.43-1.50 (1.53-1.50)	41.0-1.45 (1.49-1.45)
Unique reflections	35835 (1715)	38654 (2441)
Rmerge (%)	3.9 (31.9)	13.1 (145.4)
Rmeas (%)	7.0 (63.9)	14.6 (164.4)
Redundancy	2.2 (2.1)	5.3 (3.5)
Completeness overall(%)	92.2 (87.9)	90.1 (77.6)
<I/σ(I)>	11.9 (2.4)	11.32 (1.39)
CC (1/2)	0.989 (0.410)	0.996 (0.421)
**Refinement statistics**		
Resolution range(Å)	41.43-1.50	41.00-1.45
Rfactor (%)	14.04	17.24
Rfree(%)	19.97	19.32
r.m.s.d. bonds(Å)	0.0137	0.0117
r.m.s.d. angles (°)	1.9105	1.7354
**Ramachandran statistics** (%)		
Most favored	97.7	98.0
additionally allowed	2.3	2.0
outlier regions	0.0	0.0
**Average B factor ($Å^2$)**		
All atoms	18.359	17.959
Inhibitors	34.326	54.403
Solvent	28.685	25.601

Figure S1:

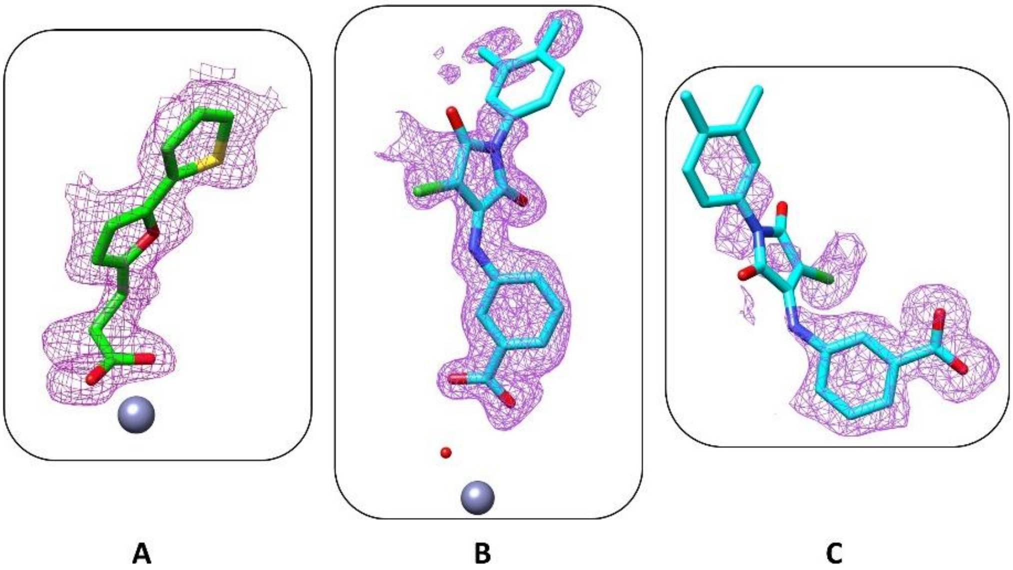

Electron density of inhibitors **1** (**A**, green) and **2** (**B**, cyan) bound to zinc (grey) and out of active site (**C**) in hCA II active site. $2F_o$-F_c maps and contoured to the 1.0 σ level.
